# The tumor suppressor Scrib interacts with the zyxin-related protein LPP, which shuttles between cell adhesion sites and the nucleus

**DOI:** 10.1186/1471-2121-6-1

**Published:** 2005-01-13

**Authors:** Marleen MR Petit, Sandra MP Meulemans, Philippe Alen, Torik AY Ayoubi, Erik Jansen, Wim JM Van de Ven

**Affiliations:** 1 Laboratory for Molecular Oncology, Department of Human Genetics, University of Leuven & Flanders Interuniversity Institute for Biotechnology (VIB), Herestraat 49, B-3000 Leuven, Belgium

## Abstract

**Background:**

At sites of cell adhesion, proteins exist that not only perform structural tasks but also have a signaling function. Previously, we found that the Lipoma Preferred Partner (LPP) protein is localized at sites of cell adhesion such as focal adhesions and cell-cell contacts, and shuttles to the nucleus where it has transcriptional activation capacity. LPP is a member of the zyxin family of proteins, which contains five members: ajuba, LIMD1, LPP, TRIP6 and zyxin. LPP has three LIM domains (zinc-finger protein interaction domains) at its carboxy-terminus, which are preceded by a proline-rich pre-LIM region containing a number of protein interaction domains.

**Results:**

To catch the role of LPP at sites of cell adhesion, we made an effort to identify binding partners of LPP. We found the tumor suppressor protein Scrib, which is a component of cell-cell contacts, as interaction partner of LPP. Human Scrib, which is a functional homologue of Drosophila *scribble*, is a member of the leucine-rich repeat and PDZ (LAP) family of proteins that is involved in the regulation of cell adhesion, cell shape and polarity. In addition, Scrib displays tumor suppressor activity. The binding between Scrib and LPP is mediated by the PDZ domains of Scrib and the carboxy-terminus of LPP. Both proteins localize in cell-cell contacts. Whereas LPP is also localized in focal adhesions and in the nucleus, Scrib could not be detected at these locations in MDCKII and CV-1 cells. Furthermore, our investigations indicate that Scrib is dispensable for targeting LPP to focal adhesions and to cell-cell contacts, and that LPP is not necessary for localizing Scrib in cell-cell contacts. We show that all four PDZ domains of Scrib are dispensable for localizing this protein in cell-cell contacts.

**Conclusions:**

Here, we identified an interaction between one of zyxin's family members, LPP, and the tumor suppressor protein Scrib. Both proteins localize in cell-cell contacts. This interaction links Scrib to a communication pathway between cell-cell contacts and the nucleus, and implicates LPP in Scrib-associated functions.

## Background

At the heart of structural and functional integrity of multicellular entities is the ability of each and every cell of it to successfully integrate signals arising from soluble factors, cell-substratum adhesion and cell-cell adhesion [1]. Correct processing of these signals allows appropriate cellular growth, differentiation, and tissue morphogenesis, but malfunctions often lie at the basis of pathologies such as tumor growth and metastasis. At sites of cell adhesion, more and more proteins are being identified that not only play a role in maintaining cell shape and motility but that, in addition to these structural functions, are also implicated in signaling events. Because of this dual function, these proteins have to interact, via multiple binding motifs, with components of both the actin cytoskeleton and signaling pathways that regulate e.g. gene expression.

**Figure 1:**
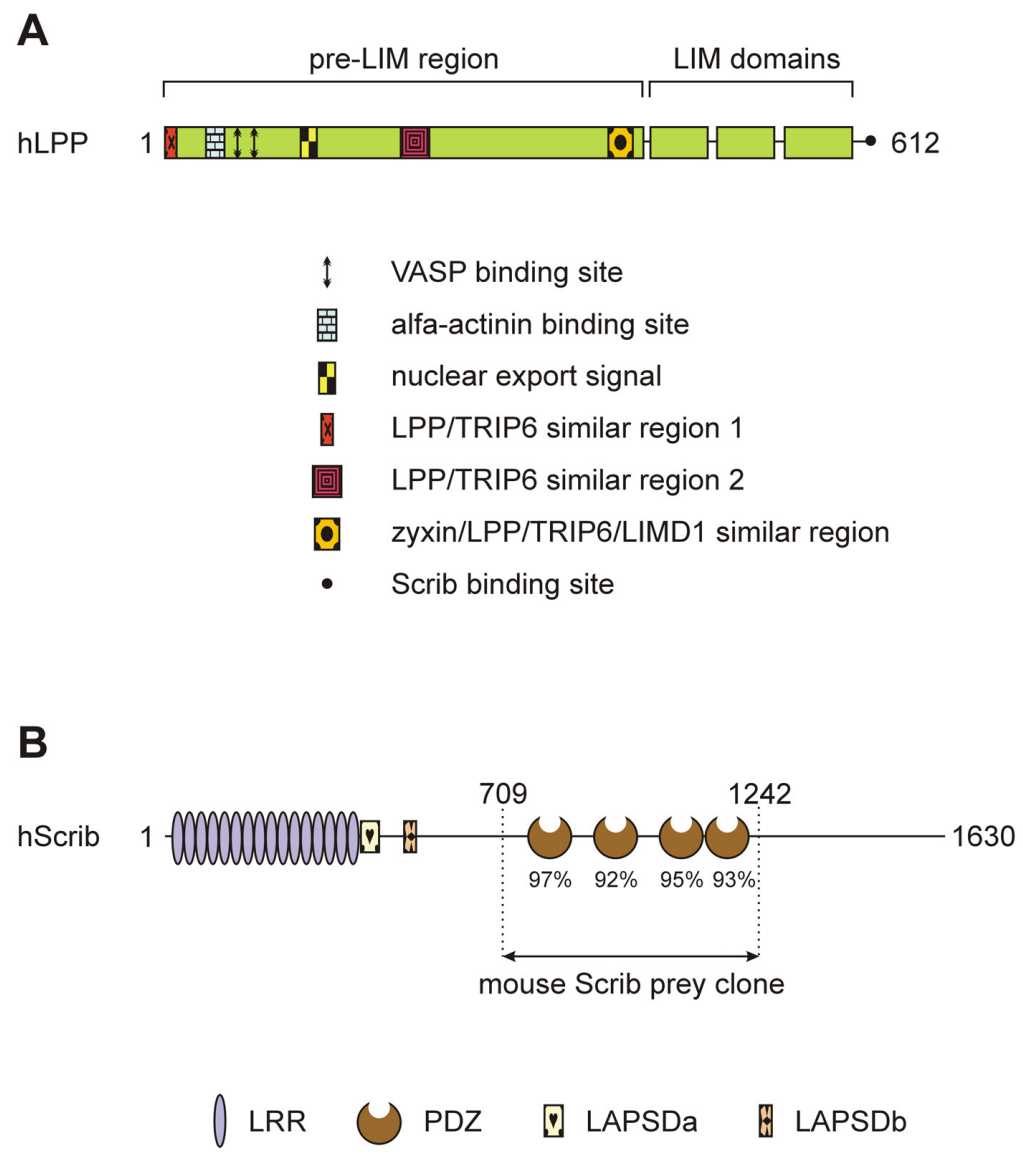
**Schematic representation of human LPP and Scrib proteins. **(A) Schematic representation of the human LPP protein. Human LPP contains a proline-rich pre-LIM region followed by three tandem LIM domains. In its pre-LIM region, LPP harbors one nuclear export signal, two VASP binding sites and one α-actinin binding site. Furthermore, LPP has two regions in common with its family member TRIP6 and one region with its family members zyxin, TRIP6 and LIMD1. At its carboxy-terminus, LPP has a binding site for Scrib. (B) Schematic representation of the human Scrib protein human Scrib is a 1630 amino acid protein that contains 16 leucine-rich repeats (LRR) followed by 2 domains that are specific for the LAP family of proteins (LAP-specific domain (LAPSD) a and b), and 4 PDZ domains. The corresponding position of the mouse Scrib prey clone that was isolated in a yeast-two hybrid screen using LPP as bait is indicated. The amino acid sequence of the PDZ domains of mouse Scrib was compared to that of human Scrib, and percentage identity is indicated for each PDZ domain.

A protein that may play a role in these processes is the LPP (Lipoma Preferred Partner) protein [2]. LPP is a member of the zyxin family of LIM domain proteins, which consists of five members: zyxin [3], TRIP6 (Thyroid Receptor Interacting Protein 6) [4], LPP [2], ajuba [5] and LIMD1 (LIM Domain containing 1) [6]. LPP has three LIM domains (zinc-finger protein interaction domains) at its carboxy-terminus, which are preceded by a proline-rich pre-LIM region containing a number of protein interaction domains (Fig. 1A). LPP has been shown to localize at sites of cell adhesion, such as focal contacts, which are membrane attachment sites to the extracellular matrix, and cell-cell contacts. However, apart from its localization in cell adhesion sites, this protein has also been shown to localize transiently in the nucleus. Because of its structural features and its characteristic to shuttle between the nucleus and the cytoplasm, LPP has been proposed to be a scaffolding protein involved in signal transduction from sites of cell adhesion to the nucleus.

Originally, we identified the *LPP *gene, as being the preferred translocation partner of the *HMGA2 *gene in a subgroup of lipomas, which are benign tumors of adipose tissue [2]. In these tumors, *HMGA2*/*LPP *fusion transcripts are expressed and identical fusion transcripts have also been found in a subgroup of pulmonary chondroid hamartomas [7], in a parosteal lipoma [8], and in a soft tissue chondroma [9]. In a case of acute monoblastic leukemia, the *LPP *gene acts as translocation partner of the *MLL *gene and the tumor expresses *MLL*/*LPP *fusion transcripts [10]. All tumor-specific fusion transcripts that are expressed in the above mentioned tumors encode similar LPP fusion proteins containing AT-hooks (DNA binding domains) of the HMGA2 or MLL proteins followed by LIM domains of LPP. These fusion proteins are mainly expressed in the nucleus [11].

At cell adhesions, the LPP protein interacts with α-actinin and VASP (vasodilator-stimulated phosphoprotein) via its pre-LIM region that contains an α-actinin binding site located near its N-terminus and two VASP-binding ("FP_4_")-motifs (Fig. 1A) [11,12]. In the nucleus, LPP has transcriptional activation capacity in reporter gene assays suggesting that it is involved in the regulation of gene expression [11]. The nucleocytoplasmic distribution of this protein involves a nuclear export signal (NES) that also resides in the pre-LIM region (Fig. 1A) [11]. Recently, we have shown that the LIM domains of LPP cooperate to target the protein to focal adhesions, and that the linker between LIM domains 1 and 2 plays a pivotal role in this targeting [13]. When overexpressed in the cytoplasm of cells, these LIM domains deplete endogenous LPP and vinculin from focal adhesions suggesting a role for LPP in focal adhesion assembly [13]. Recently, LPP was found to be highly expressed in smooth muscle [14,15], and a role for LPP in regulating cell motility was proposed [14].

In an effort to learn more about the molecular function of LPP, we performed a yeast two-hybrid screening experiment to identify potential LPP-interacting proteins. Here, we report that LPP interacts with Scrib, a member of the LAP (leucine-rich repeat and PDZ domain) family of proteins [16]. Scrib is a functional homologue [17] of *Drosophila *Scribble, a tumor suppressor that plays a role in the regulation of cellular adhesion, cell shape and polarity [18,19]. In follow-up of the results of the yeast two-hybrid screening, we have performed various experiments to find out whether the observed interaction also occurs in mammalian cells and have substantiated this interaction in vitro and in vivo. Furthermore, we have studied whether or not the Scrib protein plays a role in the subcellular targeting of LPP.

## Results

### Screening for LPP-interacting proteins by yeast two-hybrid

In a previous study [13], we showed that the LIM domains of LPP are the major units for targeting LPP to focal adhesions. LIM domains are cysteine- and histidine-rich domains that form two zinc fingers capable of mediating protein-protein interactions [20,21]. However, the protein(s) that is/are responsible for the targeting of LPP to focal adhesions, i.e. protein(s) that bind(s) to the LIM domains of LPP, are not yet known. To identify protein binding partners of the LIM domains of LPP, we performed a yeast two-hybrid screening experiment. We made use of a yeast two-hybrid system that is based on transcriptional activation of two reporter genes *HIS3 *and *LacZ *whose expression is driven by upstream GAL4 DNA-binding sites. Because all three LIM domains of LPP cooperate to target LPP to focal adhesions [13], we initially focused on a screening using a bait that contained all three LIM domains. Unlike in mammalian cells, where we have shown that the three LIM domains of LPP have transcriptional activation capacity [11], this bait, although well expressed, did not activate the reporter genes in yeast cells (results not shown). This is similar to what has been found for zyxin's LIM domains [22], but in contrast to what has been found for the three LIM domains of TRIP6 that do activate reporter genes in yeast [22]. However, the bait containing all three LIM domains of LPP appeared to be very sticky since thousands of yeast colonies were obtained in which both reporter genes were activated. In an effort to reduce background activity, we deleted the first LIM domain, or the first and the second LIM domain, in the bait, leaving the two most carboxy-terminal, or the most carboxy-terminal LIM domain(s) intact, respectively. These deletions completely abolished all background activity making these baits the baits of choice to perform a library screening. Here, we report about the screening that was performed with the bait containing only the most carboxy-terminal LIM domain of LPP. As described before [13], we showed that the third LIM domain of LPP only has a very weak targeting capacity for focal adhesions. This makes it very unlikely that, by using this bait, we would pick up a protein that targets LPP to these structures, which was the initial goal of our studies. Indeed, our screening did not reveal any focal adhesion binding partners of LPP, however, in stead, we found another very interesting LPP-interacting protein as will be outlined in the following sections.

A mouse embryonal cDNA library was screened using a bait (pGBT9-LPP_WT_) containing the third LIM domain and carboxy-terminus of human LPP (amino acids 531–612). Among ~1.0 × 10^6 ^yeast cotransformants (Leu^+ ^and Trp^+^), 56 clones were His^+ ^of which 23 were LacZ^+ ^too. PCR analysis of these His^+^/LacZ^+ ^clones, using prey-specific insert-flanking primers, revealed that 21 of the 23 obtained clones, contained a prey-construct having a 2 kb cDNA insert (results not shown). Subsequent fragmentation of the obtained 2 kb PCR products, representing the cDNA inserts of the prey-constructs, using the HaeIII restriction enzyme (frequent cutter), indicated that all 21 isolated prey-constructs, having a 2 kb insert, were identical. The 2 kb cDNA insert of one representative prey-construct was completely sequenced and the sequence was submitted to the NCBI database (Genbank accession no. AF271735).

A BLAST (Basic Local Alignment Search Tool)-search revealed that this mouse prey-construct encoded an amino- and carboxy-terminally truncated protein comprising four PDZ domains that was almost identical to the human Scrib protein (Fig. 1B), indicating that the prey-construct represented mouse Scrib. The Scrib protein contains a set of 16 leucine-rich repeats (LRRs) near its amino-terminus and four PDZ (PSD-95, Discs large, ZO-1) domains distributed throughout the remainder of the protein (Fig. 1B). The partial mouse Scrib protein, expressed by the prey-construct, corresponded to amino acids 709 – 1242 of human Scrib (Fig. 1B).

Further analysis indicated that the isolated prey-construct, which was named pACT2-mScrib, activated the *HIS3 *and *LacZ *reporter genes of the yeast only in the presence of pGBT9-LPP_WT_, identifying pACT2-mScrib as a true positive (Table 1, upper three rows).

**Table 1:** Interaction of LPP with Scrib in the yeast two-hybrid system

BAIT	PREY	HIS	LACZ
pGBT9	pACT2-mScrib	-	-
pGBT9-LPP_WT_	pACT2	-	-
pGBT9-LPP_WT_	pACT2-mScrib	+	+
pGBT9-LPP_S609A_	pACT2	-	-
pGBT9-LPP_S609A_	pACT2-mScrib	+	+
pGBT9-LPP_T610A_	pACT2	-	-
pGBT9-LPP_T610A_	pACT2-mScrib	-	-
pGBT9-LPP_D611A_	pACT2	-	-
pGBT9-LPP_D611A_	pACT2-mScrib	+	+
pGBT9-LPP_L612A_	pACT2	-	-
pGBT9-LPP_L612A_	pACT2-mScrib	-	-

Yeast cells (CG-1945), cotransformed with a bait and a prey as indicated, were selected on medium containing 5 mM 3-AT, lacking Trp, Leu and His. Yeast colonies were tested for the expression of β-galactosidase.

+ indicates strong positive interaction; - indicates no interaction.

### LPP binds to the PDZ domains of Scrib via its C-terminal tail

Since the pACT2-mScrib prey-construct contained four PDZ domains, and since PDZ domains are one of the most commonly found protein-protein interaction domains in organisms from bacteria to humans [23], it was most likely that Scrib would bind to LPP via its PDZ domains. The LPP-bait that was used to screen the library was pGBT9-LPP_WT _containing the third LIM domain and carboxy-terminus of human LPP. Although PDZ domains have been shown to bind LIM domains [24], binding to carboxy-terminal peptides appears to be the typical mode of interaction [25]. The common structure of PDZ domains comprises six β strands (βA-βF) and two α helices (αA and αB), which fold in an overall six-stranded β sandwich [25]. The binding specificity of PDZ domains is critically determined by the interaction of the first residue of helix α B (position αB1) and the side chain of the -2 residue of the C-terminal ligand. This forms the basis for PDZ classification [25]. Since all four PDZ domains of Scrib contain a histidine at position αB1, they are classified as class I PDZ domains. Therefore, based on what has been demonstrated for this subclass of PDZ domains [25,26], the carboxy-terminal sequence of Scrib target proteins is predicted to require a hydrophobic amino acid (h) at the 0 (carboxy-terminus) position, and a serine (S) or threonine (T) at the -2 position.

Theoretically, the carboxy-terminus of the LPP protein, being -STDL, thus completely fulfils the criteria for binding to the PDZ domains of Scrib. To evaluate these predictions experimentally and to demonstrate that the binding of LPP to Scrib is specific, we performed yeast two-hybrid experiments using pGBT9-LPP_WT _as well as pGBT9-LPP_S609A_, pGBT9-LPP_T610A_, pGBT9-LPP_D611A _and pGBT9-LPP_L612A _as bait. The last four baits are identical to pGBT9-LPP_WT _except for a point mutation to alanine, respectively introduced at serine^609 ^(-3 position), threonine^610 ^(-2 position), aspartate^611 ^(-1 position) and leucine^612 ^(position 0). As prey, we used pACT2-mScrib. As summarized in Table 1, this alanine-scan mutant analysis identified threonine^610 ^(-2 position) and leucine^612 ^(0 position) of LPP as being essential for binding to Scrib indicating a PDZ domain-mediated specific interaction between Scrib and the carboxy-terminus of LPP.

Additional yeast two-hybrid analysis showed that LPP did not interact with Erbin, PICK1, PSD-95, Syntenin, CASK, or AF6 PDZ domains, as summarized in Table 2.

**Table 2:** Interaction of LPP with PDZ domains of proteins different from Scrib

BAIT	PREY	HIS	-HIS
pGBT9-LPP_WT_	Scrib-PDZ	+	+
	Erbin-PDZ	+	-
	PICK1-PDZ	+	-
	PSD95-PDZ	+	-
	Syntenin-PDZ	+	-
	CASK-PDZ	+	-
	AF6-PDZ	+	-

Yeast cells, cotransformed with pGBT9-LPP_WT _and a pACT2-prey as indicated, were selected on medium lacking Leu and Trp, and either containing His or no His with 5 mM 3-AT.

+ indicates growth of yeast transformants; - indicates no growth of yeast transformants.

### LPP interacts with Scrib PDZ domains in mammalian cells

**Figure 2:**
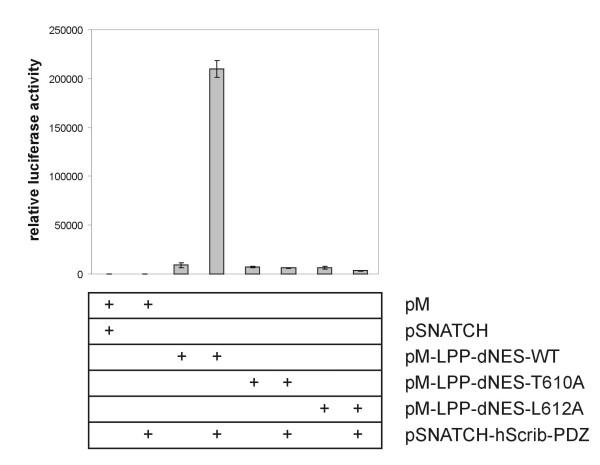
**Scrib interacts with LPP in mammalian cells. **pM-bait- and pSNATCH-prey-constructs were cotransfected into 293 cells in the combination indicated, together with a GAL4-regulated luciferase reporter and a CMV-β-galactosidase internal control. Cell lysates were assayed for luciferase activity 18–24 hours after transfection. Relative luciferase activity is reported as the average of three independent duplo experiments (with standard error).

We verified the Scrib-LPP interaction, which was identified in yeast cells, in mammalian two-hybrid experiments. Doing the assay in mammalian cells rather than in yeast cells, provides a more physiological environment: proteins are more likely to be in their physiological configuration, i.e. appropriately folded and modified posttranslationally, etc. Interaction between bait- and prey-proteins in a mammalian two-hybrid assay takes place in the nucleus. For an accurate performance of this assay, this means that bait- and prey-proteins should be localized in the nucleus. In contrast to the yeast assays, where we used partial bait-proteins, we wanted to use full length bait-proteins in the mammalian assay. However, since LPP contains a nuclear export signal (NES) (amino acids 117–128) in its pre-LIM region [11], we used bait-proteins in which this NES had been deleted. To verify whether deletion of the NES in LPP induced nuclear accumulation of the bait-proteins that were used in the mammalian two-hybrid assay, we introduced wild-type and mutated LPP-bait-proteins in 293T cells. While pM-LPP_WT_, containing GAL4-fused full length wild-type human LPP with an intact NES, was excluded from nuclei, pM-LPP_dNESWT_, containing GAL4-fused full length human LPP with a deletion of the NES, was accumulating in the nuclei of the cells (results not shown). These results indicate that deletion of the NES in the LPP bait proteins used in this study indeed induce nuclear accumulation of these proteins. To perform the mammalian two-hybrid experiments, we used as baits: pM-LPP_dNESWT_, containing full length human LPP with a deletion of its NES, and pM-LPP_dNEST610A _and pM-LPP_dNESL612A_, which are identical to pM-LPP_dNESWT _except for a point mutation to alanine introduced at threonine^610 ^(position -2) and leucine^612 ^(position 0), respectively. As determined in the yeast two-hybrid assay, each of the threonine^610 ^and leucine^612 ^residues is critical for the interaction with Scrib. As prey-protein, we used pSNATCH-hScrib_PDZ _containing a part of the human Scrib protein (amino acids 669–1233) encompassing all four PDZ-domains. As summarized in Fig. 2, the interaction between wild-type full length LPP and Scrib PDZ domains resulted in high levels of luciferase reporter activity. These high levels dropped to background levels when pM-LPP_dNEST610A _or pM-LPP_dNESL612A _were used as baits in combination with Scrib as prey. The "background" levels of luciferase that were detected when pM-LPP-baits were used in combination with pSNATCH (empty prey-vector) as prey, are due to the intrinsic transcriptional activation activity of the LPP protein [11].

These results indicate that LPP binds to Scrib PDZ domains and that this binding is abolished when amino acids at position 0 or -2 are mutated.

### Development and characterization of Scrib antibodies

**Figure 3:**
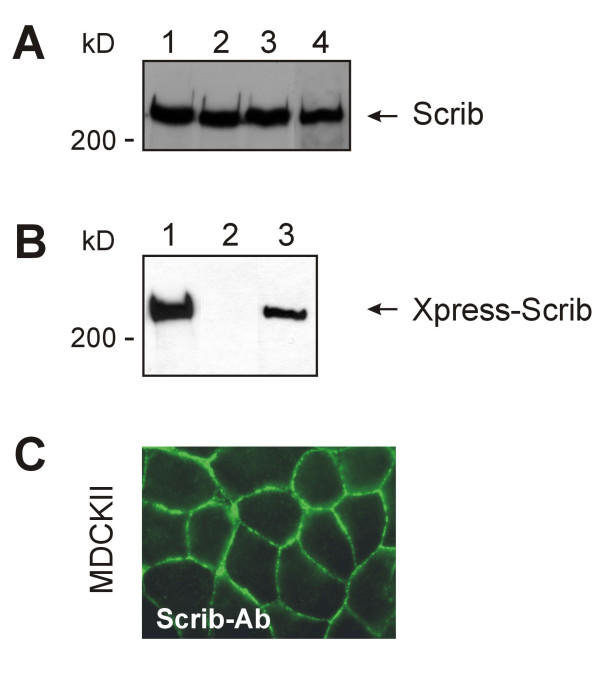
**Characterization of anti-Scrib antibodies. **(A) Total cell extracts were prepared from the following cell lines: human embryonic kidney epithelial cells (293) (lane 1), dog normal kidney epithelial cells (MDCK) (lane 2), human T lymphocytes (Jurkat) (lane 3), and African green monkey kidney fibroblast cells (CV-1) (lane 4). Approximately 30 μg of protein from each extract was analysed by SDS-PAGE and Western blotting with the Scrib-472 antibodies. The position of molecular markers are as shown. (B) Total cell extracts of 293T cells, either not transfected (lane 2), or transiently transfected with Xpress-hScrib that is composed of the full length human Scrib protein fused to an Xpress-epitope-tag at its amino-terminus (lanes 1 and 3) were analyzed by SDS-PAGE and Western blotting with an anti-Xpress antibody (lane 1) or with the Scrib-472 antibody (lanes 2 and 3). The position of molecular markers are as shown. (C) MDCKII cells, grown on glass coverslips, were fixed and stained with Scrib-472-antibodies. Immunofluorescence was visualized by epifluorescence microscopy.

To analyze expression and intracellular distribution of Scrib in cultured cells, we prepared a Scrib-specific antibody (Scrib-472), as described in the Methods section. The Scrib-472 antibody recognized a protein of an apparent molecular mass of more than 200 kDa in a number of different cell extracts (Fig. 3A). Scrib was easily detected in the epithelial cell lines 293 and MDCKII, in the fibroblast cell line CV-1, and also in the T lymphocyte cell line Jurkat (Fig. 3A). These results indicate that our antibody recognizes Scrib-proteins of different species, being human (Jurkat and 293), monkey (CV-1) and dog (MDCKII). The Scrib-472 antibody also reacted with an Xpress-hScrib fusion protein produced in 293T cells transfected with the corresponding DNA (Fig. 3B). In Fig. 3B, lane 2, which depicts a Western analysis of untransfected 293T cell lysate with Scrib-antibodies, no band of endogenous Scrib is seen. Longer exposure, however, did show a band indicating that Scrib is expressed in these cells, however, 293T cells express much lower levels of endogenous Scrib as compared to 293 cells (our unpublished observations). The Scrib protein was migrating slower in SDS gels than would be expected from its theoretically calculated molecular mass (175 kDa). Possible explanations include anomalous migration per se, and posttranslational modifications. To investigate whether the Scrib-472 antibody not only recognizes denatured Scrib protein on Western blots but also is capable of detecting Scrib in fixed cells, MDCKII cells were grown to confluency on glass coverslips and stained with the Scrib-472 antibodies. From previous studies, it is known that Scrib is localized in cell-cell contacts [17]. As shown in Fig. 3C, the Scrib-472 antibody indeed is capable to detect native Scrib in cell-cell contacts in fixed cells.

### Scrib is not localized in focal adhesions in CV-1 and MDCKII cells, and is dispensable for targeting LPP to these structures

**Figure 4:**
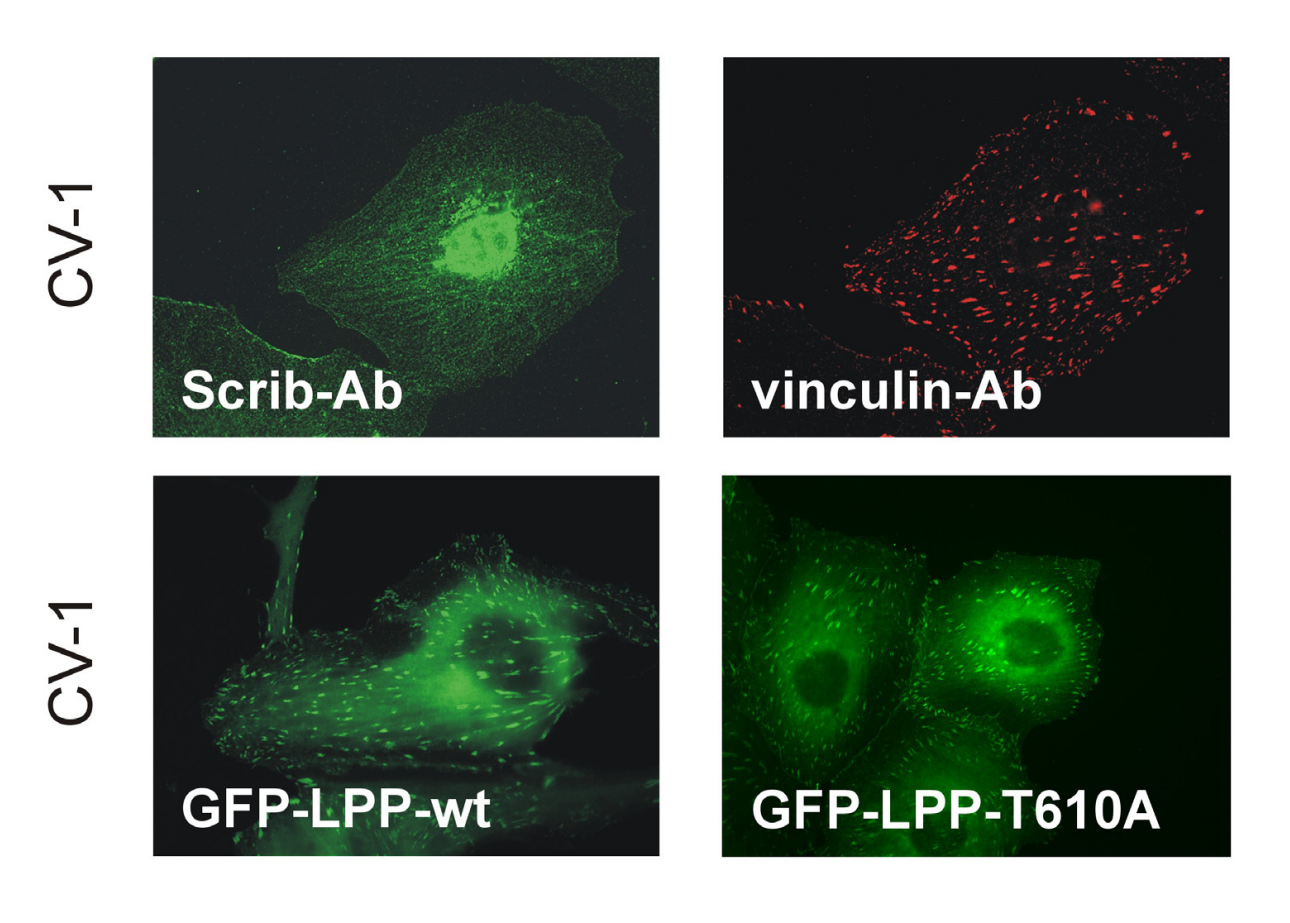
**Scrib is not localized in focal adhesions in CV-1 cells, and is dispensable for targeting LPP to these structures. **Upper panels: CV-1 cells, grown on glass coverslips, were double labelled with Scrib-472 antibodies (left panel) and anti-vinculin antibodies (right panel) used as a marker for focal adhesions. Lower panels: CV-1 cells were transiently transfected with wild-type human LPP (left panel), or LPP with a mutated carboxy-terminus (T610A) (right panel), as GFP-fusions. GFP-fluorescence was visualized by epifluorescence microscopy.

**Figure 5:**
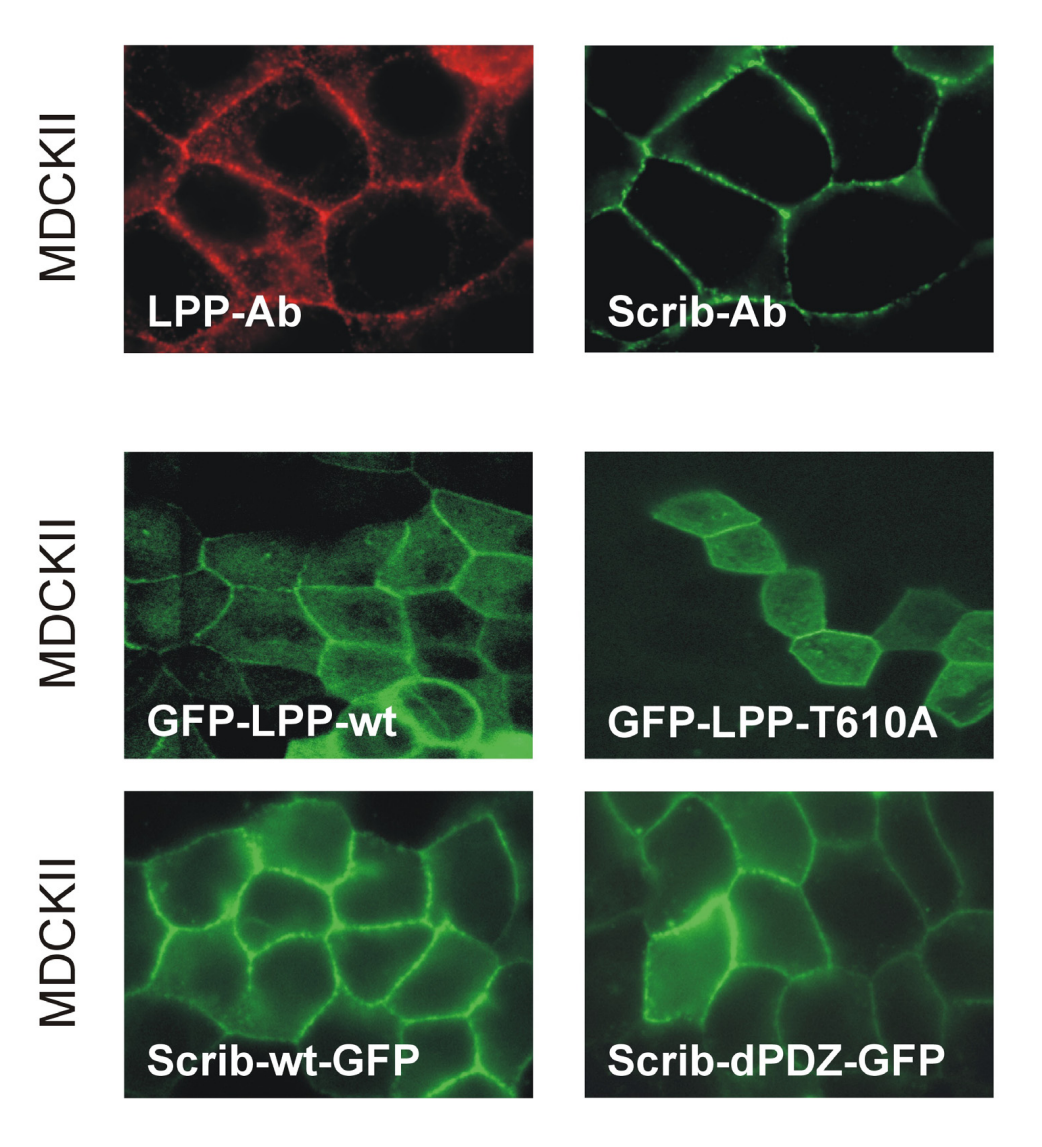
**Scrib and LPP are localized in cell-cell contacts but are dispensable for targeting each other to these structures. **Upper panels: MDCKII cells, grown on glass coverslips, were double labelled with anti-LPP antibodies (left panel) and anti-Scrib antibodies (right panel). Lower panels: MDCKII stable cell lines, expressing GFP-fusion proteins containing wild-type human LPP (upper left panel), LPP with a mutated carboxy-terminus (T610A) (upper right panel), human wild-type Scrib (lower left panel), or Scrib with a deletion of all its PDZ domains (lower right panel), were grown on glass coverslips (Scrib) or on Transwell-Clear polyester membranes (LPP). GFP-fluorescence was visualized by epifluorescence microscopy (Scrib) or by confocal microscopy (LPP).

We have shown before that LPP is localized in cell-cell contacts [11] and also for human Scrib, it was shown that it is localized in these structures [17] (also shown in Fig. 3C and 5, upper right panel). Since LPP is not only localized in cell-cell contacts but also in focal adhesions [11,13], we investigated whether also Scrib had the ability to localize at these structures. For this purpose, we used two different cell lines: the epithelial cell line MDCKII and the fibroblast cell line CV-1. However, in contrast to LPP, Scrib could not be detected in focal adhesions as shown by staining CV-1 cells with Scrib-472 antibodies (Fig. 4, upper left panel). Identical results were obtained in MDCKII cells (results not shown). Focal adhesions were indeed present, as these structures could be stained using vinculin antibodies used as a marker for focal adhesions (CV-1 cells: Fig. 4, upper right panel; MDCKII cells: results not shown). If Scrib had been present in focal adhesions, we would have detected it there, because, as shown in Fig. 3A, Scrib is highly expressed in CV-1 as well as in MDCKII cells, and as shown in Fig. 3C, Scrib-472 antibodies are able to detect Scrib in its native conformation in fixed cells. Moreover, a hScrib-GFP protein expressed in CV-1 or MDCKII cells was never detected in focal adhesions (results not shown) but was localized in cell-cell contacts (MDCKII cells: Fig. 5, lower left panel). The nature of the nuclear staining observed in CV1-cells stained with the Scrib-472 antibody (Fig. 4, upper left panel) is aspecific, as it is also obtained with the corresponding pre-immuneserum. In addition, nuclear staining was never obtained when an hScrib-GFP protein was transiently overexpressed in these cells (results not shown). Nuclear staining was also not detected in MDCKII cells as shown in Fig. 3C and 5, upper right panel. These results indicate that, in contrast to LPP, which is localized both in focal adhesions and in cell-cell contacts in CV-1 and MDCKII cells, Scrib is only localized in cell-cell contacts but not in focal adhesions in these cells.

As deduced from these results, we hypothesized that Scrib was not involved in targeting LPP to focal adhesions. Indeed, evidence for this hypothesis was obtained by transfecting CV-1 cells with a construct expressing GFP-LPP_WT _containing full length wild-type LPP, or GFP-LPP_T610A_, which is identical to GFP-LPP_WT _except for a point mutation to alanine introduced at threonine^610^, which abolishes binding to Scrib. No difference in focal adhesion localization could be detected between wild-type and mutated GFP-LPP fusion proteins (Fig. 4, lower panels).

### Scrib and LPP are dispensable to target each other to cell-cell contacts

Since Scrib and LPP both localize in cell-cell contacts [11,17] (Fig. 5, upper panels), we investigated whether Scrib was responsible for targeting LPP to cell-cell contacts. For this, we made stable MDCKII cell lines expressing wild-type and mutated GFP-coupled forms of the LPP protein, of which the mutant form is not able to bind anymore to Scrib. However, as shown in Fig. 5, lower panels, LPP proteins that could not bind to Scrib anymore were still able to localize in cell-cell contacts in a similar way as their wild-type counterparts. These results indicate that Scrib is not responsible for targeting LPP to cell-cell contacts.

We next investigated whether LPP was responsible for targeting Scrib to cell-cell contacts. To look into this aspect, we made stable MDCKII cell lines expressing either wild-type full length Scrib-GFP or a mutated Scrib-GFP protein lacking all four PDZ domains (deletion of amino acids 725–1227). However, both the full length Scrib-GFP protein as well as the mutated form lacking all four PDZ domains localized equally well in cell-cell contacts (Fig. 5, lower panels). These results indicate that the PDZ domains of Scrib are dispensable for targeting the protein to cell-cell contacts, and as a consequence LPP is not necessary to locate Scrib in cell-cell contacts.

In summary, these results indicate that LPP and Scrib are dispensable to target each other to cell-cell contacts.

### There is a direct interaction between the carboxy-terminus of LPP and the PDZ domains of Scrib

**Figure 6:**
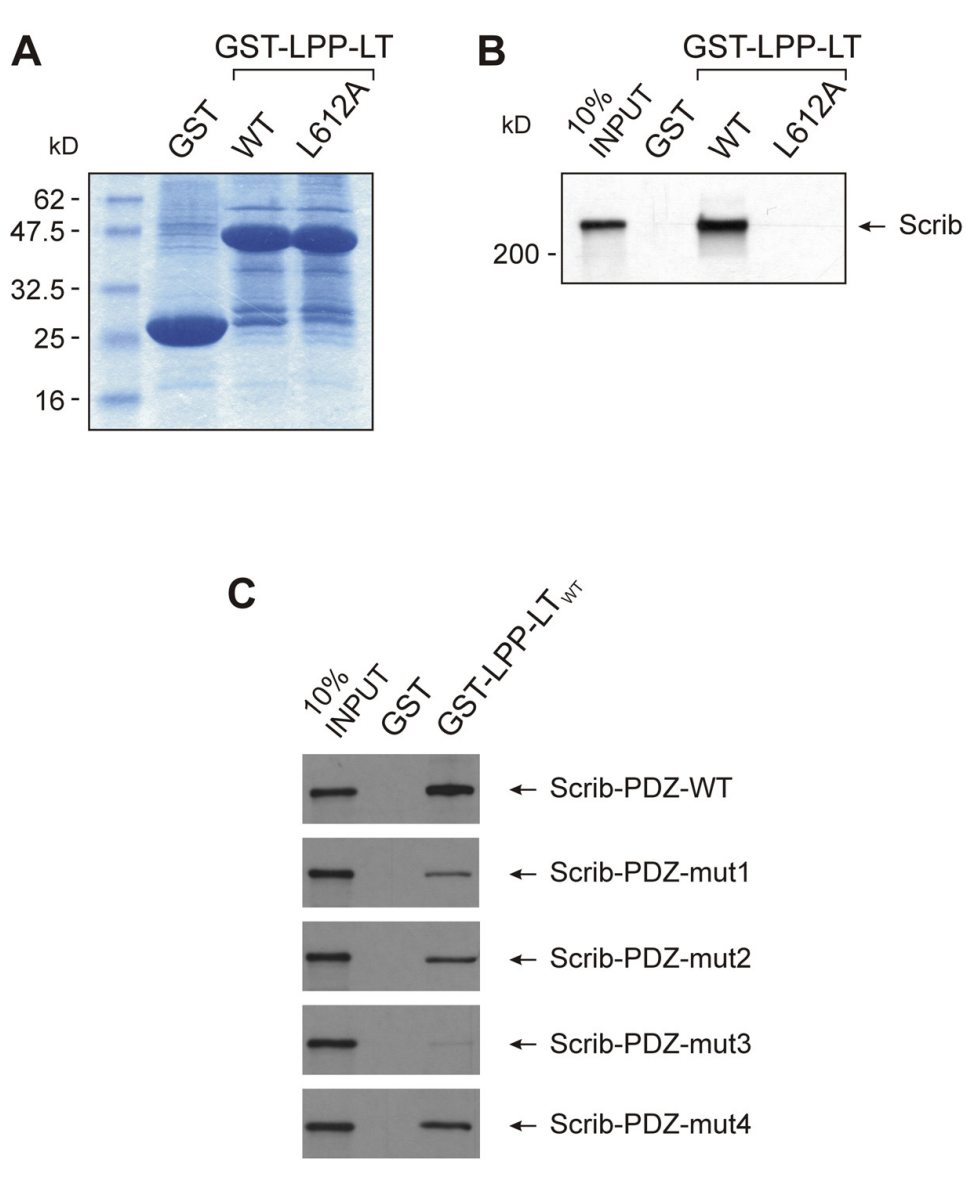
**Direct interaction between the carboxy-terminus of LPP and the PDZ domains of Scrib. **(A) GST fused to either wild-type LPP (40 amino acids of the pre-LIM region, the three LIM domains and the tail), or a similar LPP molecule with a mutated carboxy-terminus (L612A) and GST alone were expressed in *E. coli*, purified and analyzed by SDS-PAGE and Coomassie Blue staining. All proteins were expressed well. Protein markers are as indicated. (B) In vitro synthesized [^35^S]-methionine-labelled full length Scrib was incubated with immobilized GST or with either one of the above-described GST fusion proteins and allowed to interact over night at 4°C. After extensive washing, bound proteins were eluted in sample buffer, separated by SDS-PAGE and visualized by autoradiography. The amount of synthesized protein loaded as a reference on the gel corresponds to 10% of the input used in each binding experiment. (C) All four PDZ domains of Scrib (amino acids 616 – 1490), either wild-type or mutated as indicated, were synthesized in vitro and [^35^S]-methionine-labelled. these labelled proteins were incubated with immobilized GST or with GST-LPP-LT_WT _and allowed to interact over night at 4°C. Bound proteins were eluted in sample buffer, separated by SDS-PAGE and visualized by autoradiography. The amount of synthesized protein loaded as a reference on the gel corresponds to 10% of the input used in each binding experiment.

To further assess the binding between LPP and Scrib, we investigated whether there is a direct interaction between these two proteins. For this, we performed GST pull-down experiments. In vitro translated full length Scrib was tested for binding with glutathione beads, which were coupled with GST-LPP-LT_WT_, GST-LPP-LT_L612A_, or GST alone. GST-LPP-LT_WT _contains 40 amino acids of the pre-LIM region, the three LIM domains, and the wild-type carboxy-terminal tail of human LPP. GST-LPP-LT_L612A _is identical to GST-LPP-LT_WT _except for a point mutation to alanine introduced at leucine^612 ^(position 0). All GST-fusion proteins as well as GST alone were expressed well in *E. coli *(Fig. 6A). As shown in Fig. 6B, Scrib interacted specifically with the wild-type LPP protein but not with its mutated form, GST-LPP-LT_L612A _or with GST alone. These results indicate that there is a specific and direct interaction between LPP and Scrib.

To further investigate the requirements in the Scrib protein for binding to LPP, we performed additional GST pull-down experiments. From our previously described experiments (yeast and mammalian two-hybrid), it was clear that the PDZ domains of Scrib bind to LPP. These findings were confirmed by using GST pull-down: as shown in Fig. 6C, upper panel, a portion of the Scrib protein encompassing all four PDZ domains was efficiently pulled down by GST-LPP-LT_WT_. To find out which of the four PDZ domains of Scrib was responsible for the observed interaction with LPP, we mutated the PDZ domains of Scrib, one at the time, by destroying their carboxylate binding loop (LG → AE), and tested how efficiently these mutated proteins were pulled down by GST-LPP-LT_WT_. From the results, which are presented in Fig. 6C, we can conclude that all four PDZ domains of Scrib more or less contribute to the binding to LPP, but that PDZ 3 is most important, since binding to GST-LPP-LT_WT _was almost completely abolished when the carboxylate binding loop of this PDZ domain was destroyed.

### Scrib can target LPP to an ectopic location in vivo through its PDZ domains

**Figure 7:**
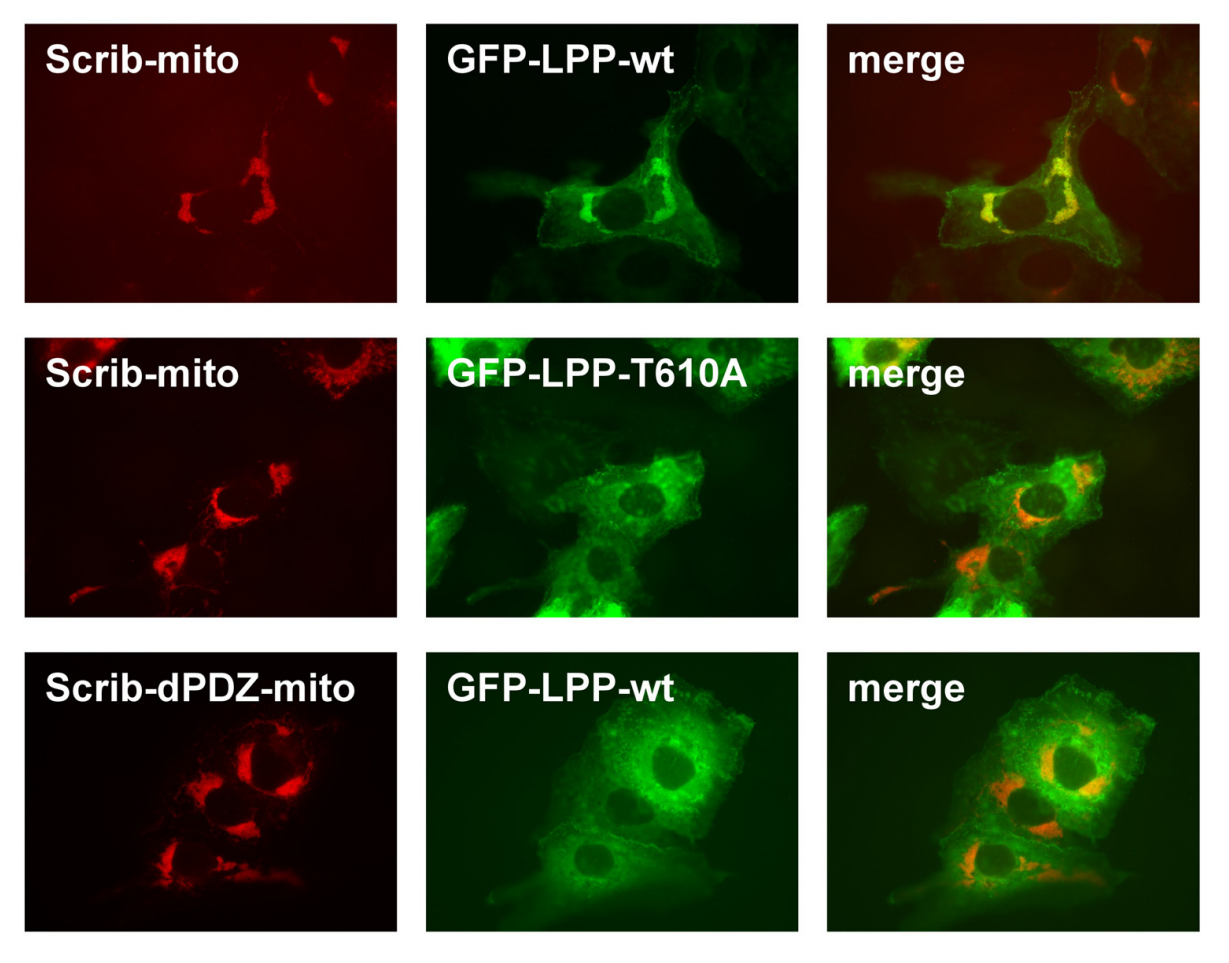
**Scrib can recruit LPP to an ectopic location in vivo through its PDZ domains. **CV-1 cells were transiently co-transfected with Xpress-hScrib-mito or Xpress-hScribdPDZ-mito, and GFP-fusions of wild-type full length human LPP, or LPP with a mutated carboxy-terminus (T610A). Xpress-hScrib-mito and Xpress-hScribdPDZ-mito are composed of the human full length Scrib protein with or without its PDZ domains, respectively, which is fused to an Xpress-epitope-tag at its amino-terminus, and to an ActA-derived mitochondrial membrane anchor at its carboxy-terminus, Cells were stained with an anti-Xpress antibody to detect Xpress-Scrib(dPDZ)-mito. Immunofluorescence and GFP were visualized by epifluorescence microscopy. The focal adhesion localization of the GFP-LPP proteins is not visible in these pictures because a focal plane corresponding to mitochondrial staining is shown.

Evidence for an in vivo interaction between Scrib and LPP was obtained by performing mitochondrial targeting experiments. We tested if Scrib was sufficient to recruit LPP to an ectopic location in living cells. The membrane anchor of the ActA sequence has been shown previously to be sufficient to target proteins expressed in mammalian cells to the surface of mitochondria [27,28]. This ectopic localization allows testing ligand recruitment in vivo. For this purpose, we generated a chimera named Xpress-hScrib-mito made up by an Xpress-epitope tag fused to the amino-terminus of human full length Scrib and linked in frame to the membrane anchor of the *Listeria monocytogenes *protein ActA (mito). Expression of this construct was confirmed by Western blotting with the use of an anti-Xpress antibody (results not shown). CV-1 cells were transiently transfected with Xpress-hScrib-mito and full length wild-type or carboxy-terminally mutated LPP green fluorescent protein fusions. Cells were stained with an anti-Xpress antibody and examined by fluorescence microscopy. In all transfected cells, the Xpress-hScrib-mito chimera localized to mitochondria, as shown in Fig. 7, left upper and middle panels. As shown in Fig. 7, upper right panels, wild-type LPP can be recruited to Xpress-hScrib-mito on mitochondria. This recruitment of LPP to Xpress-Scrib-mito-coated mitochondria was completely abolished when the carboxy-terminus of LPP was mutated (Fig. 7, middle panels).

To investigate the importance of the PDZ domains of Scrib in this recruitment of LPP, we deleted all four PDZ domains (amino acids 724–1192) from Xpress-hScrib-mito (=Xpress-hScribdPDZ-mito) and tested whether this PDZ-less protein still was able to recruit LPP to mitochondria. As shown in Fig. 7, lower panels, Xpress-hScribdPDZ-mito lost its ability to recruit LPP to mitochondria. These results indicate that Scrib can recruit LPP to an ectopic location in vivo, and that the PDZ domains of Scrib are an absolute requirement for this activity.

As mentioned above, the Xpress-hScrib-mito chimera localized to mitochondria in all cells that expressed this protein. However, LPP, which was co-expressed, was only recruited to mitochondria in a small fraction of these cells. This issue will be further addressed in the Discussion section.

## Discussion

In the course of our studies of chromosomal aberrations in benign tumors, we have previously discovered the *LPP *gene as being rearranged in certain subtypes of these tumors [2], and identified the LPP protein as a member of the zyxin family of proteins [11]. In this study, we report that LPP specifically interacts with Scrib. We provide evidence that this interaction is mediated by the carboxy-terminus of LPP on the one hand, and the PDZ domains of Scrib on the other hand. Futhermore, we show that Scrib is not necessary for targeting LPP to focal adhesions, and that Scrib and LPP are dispensable to target each other to cell-cell contacts.

Scrib is a member of the LAP (LRR (leucine-rich repeat) and PDZ (PSD-95/Discs-large/ZO-1)) family of membrane-associated proteins that play a role in the regulation of cell polarity [16]. LAP family members have been identified in mammals (Erbin, Densin-180, Lano, and Scrib) [29-32], in *Caenorhabditis elegans *(LET-413) [33], and in *Drosophila melanogaster *(Scribble) [18]. LAP proteins contain a set of leucine-rich repeats (LRRs) at their amino-terminus, and either four (Scrib and Scribble), one (Erbin, Densin-180 and LET-413) or no (Lano) copies of the PDZ domain. A specific characteristic of these proteins are the LAP-specific domains (LAPSa and b), which are located carboxy-terminally of the LRRs [34].

Most information regarding the function of Scrib comes from studies in *Drosophila melanogaster*. *Drosophila *Scribble was identified as being required for the apical confinement of polarity determinants in epithelia [18]. Mutations in Scribble cause aberrant cell shapes and loss of the monolayer organization in embryonic epithelia. Scribble is localized in septate junctions and loss of Scribble function results in the misdistribution of apical proteins and adherens junctions to the basolateral cell surface. Subsequent studies in *Drosophila *provided evidence that Scribble is a tumor suppressor and cooperates with two other tumor suppressors, Lethal giant larvae (Lgl) and Discs-large (Dlg) to regulate cell polarity and growth control [19]. Recently, these three tumor suppressors were shown to regulate cell size and mitotic spindle asymmetry in *Drosophila *neuroblasts [35]. The role of Scribble in tumorigenesis was further supported by the discovery that Scribble mutants cooperate with oncogenic Ras or Notch to cause neoplastic overgrowth of the eye disc [36], and that cooperation between oncogenic Ras and inactivation of Scribble leads to metastatic behavior [37]. Additional studies in *Drosophila *implicate Scribble in the regulation of synaptic plasticity and synaptic vesicle dynamics [38,39], and show that Scribble is essential for olfactory behavior in *Drosophila *[40].

As for mammalian Scrib, little information is available at the moment. Relating to the control of cell polarity and proliferation, human Scrib was found to be a functional homologue of the *Drosophila scribble *protein [17]. Polarity defects and tumorous overgrowth of Scribble-mutant flies are rescued by human Scrib predicting an important role for human Scrib in the suppression of mammalian tumorigenesis. Further support for this hypothesis, was obtained by the fact that human and mouse Scrib are targeted for degradation by high-risk papillomavirus E6 proteins [32,41]. Human papilloma viruses cause papillomas or warts on skin, genital tissues, and the upper respiratory tract, and high-grade lesions progress to carcinomas at a high frequency. The high-risk subgroup of human papilloma viruses detected in these lesions have been causally linked to the development of over 90% of uterine cervical carcinomas, the second leading cause of cancer-related deaths among women world-wide. High-risk papilloma virus E6 proteins direct Scrib for degradation by directly binding to the PDZ-domains of Scrib.

In this regard, it is noteworthy that we observed a remarkable aspect regarding the expression levels of Scrib in a number of mammalian cell lines. As already mentioned before (Fig. 3), we noticed that 293T cells expressed much lower levels of Scrib as compared to 293 cells. 293T cells are derived from 293 but, in contrast, these cells stably express Simian Virus 40 largeT antigen. SV40 large T is a powerful oncoprotein capable of transforming a variety of cell types [42]. Its transforming activity is attributed to its binding and manipulation of the function of certain key tumor suppressors and cell regulatory proteins such as retinoblastoma and p53. However, certain factors that contribute to its full transformation potential are not yet completely understood. We hypothesize that large T induces the downregulation of Scrib expression, and that Scrib contributes to the transformation potential of SV40 large T.

In addition to its role as a tumor suppressor, Scrib was also implicated in the regulation of planar cell polarity, a role that is not established for *Drosophila *Scribble [43], and it was shown that disruption of Scrib is the causal factor for the severe neural tube defects that occur in the *circletail *mouse [44]. Disruption of neural tube closure leads to a group of disorders termed neural tube defects, which are one of the commonest causes of congenital malformation and lethality in humans. The most severe form of neural tube defect is craniorachischisis, in which almost the entire brain and spinal cord remain open. Craniorachischisis comprises 10–20% of human neural tube defects, and is caused by a failure to initiate neural tube formation at the start of neurulation. *Circletail *is one of only two mouse mutants that exhibit craniorachischisis. The fact that Scrib was identified as the gene that was mutated in this mouse attributes an important role for Scrib in development [44].

We show here that Scrib is expressed equally well in very different cell types, such as Jurkat cells, which are human T lymphocytes, epithelial cells such as 293 and MDCKII cells, and in fibroblasts such as CV-1 cells. As described above, the function of Scrib and its *Drosophila *ancestor Scribble have been mainly addressed in epithelial cells. To our knowledge, nothing is known yet about the function of Scrib in other cell types such as lymphocytes and fibroblasts.

We show here that LPP specifically binds to and partially co-localizes with Scrib in cell-cell contacts of epithelial and fibroblastic cell lines. Previous studies have shown that PDZ domain proteins play an important role in the targeting of proteins to specific membrane compartments and in the assembly of these proteins into supramolecular complexes [25]. Therefore, we investigated whether Scrib was essential to localize LPP in cell-cell contacts. However, as demonstrated by these experiments, Scrib is not necessary to target LPP to these structures. These findings are similar to what has been found for targeting of zyxin family members to focal adhesions. Recently, zyxin and TRIP6 were shown to interact with members of the p130^Cas ^family of signal transducers, which are focal adhesion components [22]. This interaction is primarily mediated by the LIM domains of zyxin and TRIP6. One specific function associated with the LIM domains of zyxin family members is targeting to focal adhesions. Despite this feature of the zyxin family LIM domains, and despite their interaction with p130Cas, it was shown that p130Cas is not required for focal adhesion localization of zyxin and TRIP6 [22]. We also investigated whether LPP was responsible for targeting of Scrib to cell-cell contacts. However, as demonstrated by our experiments, also this appeared not to be the case. In fact, our results indicate that all of the PDZ domains of Scrib are dispensable for targeting the protein to cell-cell contacts. For epithelial cells, these results are in agreement to what has been published in the course of our investigations by Legouis and Jaulin-Bastard *et al*. [45], who have shown that a point mutation of a specific proline residue that is located at position 305 in LRR number 13 of human Scrib is enough to abolish membrane localization.

Taken into account that PDZ domains vary in their range and stringency of specificity [25], it is not excluded that LPP might bind to other PDZ domains than the ones of Scrib. Concerning Scrib, to date, three other proteins have been described that bind to the PDZ domains of Scrib: as mentioned above, the high-risk human papillomavirus E6 protein [32] interacts with the PDZ domains of human Scrib, whereas the GUKH (guanylate kinase holder) protein was shown to bind to the PDZ domains of Scribble at *Drosophila *synapses [38], and very recently, mammalian Scrib was shown to form a tight complex with the βPIX exchange factor at neuronal presynaptic compartments [46]. These findings raise the possibility that different binding partners of the Scrib PDZ domains, including LPP, can compete with each other for binding to Scrib, and as such play a role in processes in which Scrib is involved.

In this regard, it is worth mentioning that the binding of LPP to Scrib appears to be regulated. In our mitochondrial targeting experiments (Fig. 7), we noticed that the full length wild-type LPP-protein was not targeted to Scrib-coated mitochondria in all cells. In fact, in the majority of these cells, full length wild-type LPP was not recruited by Scrib. We hypothesize that the binding of LPP to Scrib is regulated by an intra- or intermolecular interaction of LPP, as a result of which the carboxy-terminal tail is hidden in such a way that it is not available anymore for binding to Scrib. One piece of information that supports this hypothesis is the observation that, in contrast to full length LPP, the carboxy-terminal region containing only the LIM-domains and the tail but lacking the pre-LIM region, was efficiently recruited to Scrib-coated mitochondria in nearly 100% of the cells examined while carboxy-terminally mutated versions were not recruited (our unpublished results). Our observations are similar to what has been reported for the binding of zyxin to the tumor suppressor warts/LATS1. In in vitro binding experiments, it was demonstrated that parts of the zyxin protein containing LIM domains 1 and 2 efficiently bind to warts/LATS1 while the full length protein does not bind [47]. Based on these findings, the authors speculated that the LIM1/2 domains are masked in full-length zyxin, and that intramolecular and/or intermolecular modifications may regulate the interaction between zyxin and warts/LATS1.

## Conclusions

Taken together the fact that LPP shuttles between cell adhesion sites and the nucleus [11,48], and the evidence that we have provided here that Scrib interacts with LPP, establishes that Scrib is connected to the communication pathway between cell adhesion sites and the nucleus of which LPP is an important element, and suggests that LPP is implicated in Scrib-associated cellular events.

## Methods

### Plasmid constructs

The GFP-LPP construct was described before [11]. A construct expressing Xpress-hScrib-mito was made by cloning the coding region of human Scrib with a mutated stop codon in the pcDNA3.1/His vector (Life Technologies) followed by inserting a DNA fragment encoding the membrane anchor of ActA (LILAMLAIGVFSLGAFIKIIQLRKNN; a kind gift of Evelyne Friederich, Centre de Recherche Public-Santé, Luxembourg) behind the mutated stop codon. All amino acid changes in Scrib and LPP were made, using the QuikChange™ Site-Directed Mutagenesis Kit (Stratagene) according to the supplier's protocols. All synthetic mutations, ligation sites and PCR-amplified regions were verified by sequencing. Protein expression was checked by Western blotting.

### Construction and sequencing of a full-length human Scrib cDNA

The KIAA0147 partial cDNA clone was kindly provided by Takahiro Nagase (Kazusa DNA Research Institute, Japan). In order to obtain full-length 5'-cDNA sequences encoding human Scrib, RNA-linker mediated 5'-RACE (RLM-RACE) was performed according to published protocols [49] using RNA isolated from HEK293 cells. The RLM-anchor primer sequence is: 5'-GGGCATAGGCTGACCCTCGCTGAAA-3'. The gene-specific primers are 1) 5'-CACGTCCAGCTCCACCAGCTGCATG-3' and 2) 5'-GAAGTTGGCCACCTCGGGAGGCAAC-3' (nested). This allowed us to construct a composite cDNA of about 5.1 kb which was completely sequenced (Genbank accession no. AF240677).

### Yeast two-hybrid system

The Matchmaker Two-Hybrid System 2 was used (Clontech). All experiments were performed in the yeast reporter strain CG-1945. Bait-constructs were made using the vector pGBT9 (Clontech). The prey-constructs pACT2-AF6, pACT2-Erbin, pACT2-PICK1, and pACT2-PSD95 were kindly provided by Jean-Paul Borg (INSERM, Marseille, France), and were described in Audebert and Navarro et al., (AF6, Erbin, PICK1) [46] and in Saito et al., (PSD-95) [31]. The prey-constructs pACT2-Syntenin and pACT2-CASK were kindly provided by Pascale Zimmermann (University of Leuven & VIB, Belgium). An oligo(dT)- and randomly primed prey-cDNA library constructed with mRNA from 12.5 day embryonic mice using pACT2 as vector [50] was kindly provided by Kristin Verschueren and Danny Huylebroeck (University of Leuven & VIB, Belgium).

The prey-library was screened as follows: yeast strain CG-1945, containing a *HIS3 *and a *lacZ *reporter gene under the control of promoters containing GAL4-binding sites, was transformed with 66 μg of bait-DNA and 33 μg of prey-library-DNA using a LiAc high efficiency transformation protocol [51]. Transformants were grown for 10 days at 30°C on triple selective (lacking Trp, Leu and His) synthetic dropout (SD^---^) agar plates containing 5 mM 3-AT (Sigma).

Transformed His^+ ^yeast colonies were restreaked on new SD^--- ^agar plates and grown for another 1 to 2 days. Colony-lift filter assays were performed for the qualitative measurement of β-galactosidase activity according to standard protocols.

### Cell culture, stable cell lines and transfections

Cell lines used included CV-1 (ATCC CCL-70), HEK293 (ATCC CRL-1573), 293T (HEK 293 cells expressing the SV40 T-antigen), Jurkat (ATCC TIB-152), and MDCK strain II (Dog normal kidney epithelial cells). Jurkat cells were grown in RPMI 1640 (Life Technologies) supplemented with 10% fetal bovine serum. All other cell lines were grown in DMEM/F12 (1:1) (Life Technologies, Inc.) supplemented with 10% fetal bovine serum. Cells were cultured at 37°C in a humidified CO_2 _incubator.

Transient transfections were performed using FuGene™ 6 Transfection Reagent (Boehringer Mannheim) according to the supplier's instructions. Cells were incubated at 37°C for 18–24 hours before analysis.

Stable MDCK strain II cell lines were made expressing wild-type and carboxy-terminally mutated human GFP-LPP proteins, wild-type full length Scrib-GFP, or Scrib-GFP lacking all four PDZ-domains. Transfection of MDCK cells was performed using Lipofectamine 2000 Reagent (Life Technologies) according to the manufacturer's instructions. Transfected cells were selected in medium containing 250 μg/ml G418 (Life Technologies), and resistant colonies were isolated 10–14 days later. Individual clones were screened for expression of the respective GFP fusion proteins by Western blotting using a rabbit polyclonal anti-GFP antibody (Tebu Bio).

### Mammalian two-hybrid system

Bait-constructs were made using pM-vectors [52], prey-constructs were made in the pSNATCH-vector [53]. 24 hours upon seeding, semi-confluent HEK293 cells on 24-well plates were transiently cotransfected with 100 ng DNA of a bait-construct, 100 ng DNA of a prey-construct, 250 ng DNA of a luciferase reporter construct and 50 ng of CMV-β-galactosidase DNA (internal control for transfection efficiency). The reporter construct contains the gene encoding the firefly luciferase enzyme, which is under the control of a minimal promoter containing five consecutive GAL4-binding sequences (kindly provided by W. Schaffner and D. Escher, Zürich, Switzerland). Cell lysates were prepared 18 to 24 hours after transfection and assayed for luciferase activity as described previously [11].

### In vitro transcription/translation and GST pull-down assays

All in vitro translation reactions were carried out using the TNT T7 Quick Coupled Transcription/Translation System (Promega) following the manufacturer's instructions. For GST pull-down assays, bacterial expression constructs were made using pGEX-5X vectors (Amersham-Pharmacia Biotech) directing the synthesis of glutathione S-transferase (GST) fusion proteins containing wild-type or mutated forms of human LPP. These fusion proteins were purified according to manufacturer's instructions and verified by SDS-PAGE. GST fusion proteins or GST alone, bound to glutathione-agarose beads, were incubated with in vitro synthesized [^35^S]-methionine-labelled full length human Scrib protein, or a portion of the human Scrib protein encompassing all four PDZ domains (amino acids 616–1490) (wild-type or mutated) in NENT_100 _buffer (100 mM NaCl, 20 mM Tris-HCl pH = 7.6, 1 mM EDTA, 0.1% NP-40, protease inhibitors). This mixture was tumbled overnight at 4°C. Subsequently the beads were washed 5 times in 500 μl NENT_100 _buffer, resuspended in 25 μl SDS-PAGE sample buffer and incubated at 95°C for 5 minutes. Proteins were separated by SDS-PAGE and interacting Scrib was detected by autoradiography.

### Scrib-specific antiserum and commercial antibodies

The Scrib-specific polyclonal antiserum Scrib-472 was prepared by Eurogentec by immunization of rabbits with a keyhole limpet hemocyanin (KLH) coupled peptide ^1612^CSSRRPVRPGRRGLGPVPS^1630 ^(19 C-terminal AA of human Scrib). For the detection of endogenous LPP in MDCKII cells, a LPP-specific monoclonal antibody was used (BD Biosciences, Transduction Laboratories). For the detection of GAL4-fusion proteins in immunocytochemistry, a rabbit polyclonal anti-GAL4 DNA-binding domain antibody (Tebu Bio) was used. Vinculin was detected in cells with a monoclonal anti-vinculin antibody (Sigma, clone hVIN-1), Xpress-tagged proteins with a monoclonal anti-Xpress antibody (Life Technologies). Fluorescently-tagged Alexa-antibodies (Molecular Probes) were used as secondary antibodies for immunofluorescence detection.

### SDS-PAGE and Western blotting

Eukaryotic cell extracts were prepared by harvesting the cells in PBS (phosphate buffered saline), and subsequent lysis of the cell pellets in SDS-PAGE sample buffer (60 mM TRIS-HCl pH = 6.8, 12% glycerol, 4% SDS, 5% β-mercapto-ethanol). Protein concentrations in cell extracts were determined using BCA Protein Assay Reagent (Pierce) according to the manufacturer's instructions. 30 μg of proteins of each cell extract were loaded on 5% SDS-polyacrylamide gels. After size-separation, proteins were electrophoretically transferred to PROTEAN Nitrocellulose Membranes (Schleicher and Schuell). ECL Western blotting was performed using Western Lightning Chemiluminescence Reagent Plus (Perkin Elmer Life Sciences) according to the supplier's instructions.

### GFP-fluorescence and indirect immunocytochemistry

CV-1 or 293T cells seeded on glass coverslips, and MDCKII cells seeded on glass coverslips or on Transwell-Clear polyester membranes (0.4 μm, Costar) were fixed in 4% formaldehyde for 20 minutes at room temperature followed by three washes in PBS containing 0.1 mM CaCl_2 _and 0.1 mM MgCl_2 _(PBS^++^). For GFP-fluorescence, slides were mounted in vectashield mounting medium (Vector Laboratories, Inc.) and analyzed on a Zeiss Axiophot fluorescence microscope equipped with an RT slider SPOT camera (Diagnostic Instruments, Inc.) using SPOT RT Software v3.4, or by confocal microscopy (MRC-1024 Laser Scanning Confocal Imaging System; Bio-Rad). For indirect immunocytochemistry, after fixation, quenching was performed by incubating the cells for 10 minutes at room temperature in PBS^++ ^containing 50 mM NH_4_Cl. Cells were then permeabilized with 0.4% Triton-X-100 for 5–10 minutes at room temperature. Unspecific binding was blocked with 0.5% Blocking Reagent (Roche) in PBS^++ ^for 30 minutes at room temperature. Subsequently, the slides were incubated with primary antibodies for 1 hour at room temperature. After washing the cells three times in PBS^++^, bound primary antibodies were detected with fluorescently labelled secondary antibodies (Molecular Probes) for 30 minutes at room temperature. Following three washes in PBS^++^, slides were mounted and analyzed as described for GFP-fluorescence.

## Authors' contributions

MMRP designed the study, performed the yeast and mammalian two-hybrid experiments, as well as the mitochondrial targeting experiments, made the LPP-stable cell lines, performed the confocal microscopy with these cell lines, and prepared the manuscript. SMPM constructed most of the DNA constructs, made the Scrib-stable cell lines, performed the epifluorescence microscopy with these cell lines, and carried out the Western blotting experiments. PA performed the GST pull down experiments, and contributed to the characterization of the Scrib-472 antibodies. TAYA contributed to the establishment of a full length Scrib cDNA clone, and to the writing of the manuscript. EJ contributed to the development of the Scrib-472 antibodies, to the establishment of a full length Scrib cDNA clone, and to the funding of the project. WJMVDV contributed to the writing of the manuscript and to the funding of the project.

## References

[B1] Aplin AE, Howe AK, Juliano RL (1999). Cell adhesion molecules, signal transduction and cell growth. Curr Opin Cell Biol.

[B2] Petit MMR, Mols R, Schoenmakers EF, Mandahl N, Van de Ven WJM (1996). Lpp, the preferred fusion partner gene of hmgic in lipomas, is a novel member of the lim protein gene family. Genomics.

[B3] Beckerle MC (1986). Identification of a new protein localized at sites of cell-substrate adhesion. J Cell Biol.

[B4] Yi J, Beckerle MC (1998). The human trip6 gene encodes a lim domain protein and maps to chromosome 7q22, a region associated with tumorigenesis. Genomics.

[B5] Goyal RK, Lin P, Kanungo J, Payne AS, Muslin AJ, Longmore GD (1999). Ajuba, a novel lim protein, interacts with grb2, augments mitogen-activated protein kinase activity in fibroblasts, and promotes meiotic maturation of xenopus oocytes in a grb2- and ras-dependent manner. Mol Cell Biol.

[B6] Kiss H, Kedra D, Yang Y, Kost-Alimova M, Kiss C, O'Brien KP, Fransson I, Klein G, Imreh S, Dumanski JP (1999). A novel gene containing lim domains (limd1) is located within the common eliminated region 1 (c3cer1) in 3p21.3. Hum Genet.

[B7] Rogalla P, Kazmierczak B, Meyer-Bolte K, Tran KH, Bullerdiek J (1998). The t(3;12)(q27;q14-q15) with underlying hmgic-lpp fusion is not determining an adipocytic phenotype. Genes Chromosomes Cancer.

[B8] Petit MMR, Swarts S, Bridge JA, de Ven WJM (1998). Expression of reciprocal fusion transcripts of the hmgic and lpp genes in parosteal lipoma [in process citation]. Cancer Genet Cytogenet.

[B9] Dahlen A, Mertens F, Rydholm A, Brosjo O, Wejde J, Mandahl N, Panagopoulos I (2003). Fusion, disruption, and expression of hmga2 in bone and soft tissue chondromas. Mod Pathol.

[B10] Daheron L, Veinstein A, Brizard F, Drabkin H, Lacotte L, Guilhot F, Larsen CJ, Brizard A, Roche J (2001). Human lpp gene is fused to mll in a secondary acute leukemia with a t(3;11) (q28;q23). Genes Chromosomes Cancer.

[B11] Petit MMR, Fradelizi J, Golsteyn RM, Ayoubi TAY, Menichi B, Louvard D, Van de Ven WJM, Friederich E (2000). Lpp, an actin cytoskeleton protein related to zyxin, harbors a nuclear export signal and transcriptional activation capacity. Mol Biol Cell.

[B12] Li B, Zhuang L, Reinhard M, Trueb B (2003). The lipoma preferred partner lpp interacts with alpha-actinin. J Cell Sci.

[B13] Petit MMR, Meulemans SMP, Van De Ven WJM (2003). The focal adhesion and nuclear targeting capacity of the lim-containing lipoma-preferred partner (lpp) protein. J Biol Chem.

[B14] Gorenne I, Nakamoto RK, Phelps CP, Beckerle MC, Somlyo AV, Somlyo AP (2003). Lpp, a lim protein highly expressed in smooth muscle. Am J Physiol Cell Physiol.

[B15] Nelander S, Mostad P, Lindahl P (2003). Prediction of cell type-specific gene modules: Identification and initial characterization of a core set of smooth muscle-specific genes. Genome Res.

[B16] Bilder D, Birnbaum D, Borg JP, Bryant P, Huigbretse J, Jansen E, Kennedy MB, Labouesse M, Legouis R, Mechler B, Perrimon N, Petit MMR, Sinha P (2000). Collective nomenclature for lap proteins. Nat Cell Biol.

[B17] Dow LE, Brumby AM, Muratore R, Coombe ML, Sedelies KA, Trapani JA, Russell SM, Richardson HE, Humbert PO (2003). Hscrib is a functional homologue of the drosophila tumour suppressor scribble. Oncogene.

[B18] Bilder D, Perrimon N (2000). Localization of apical epithelial determinants by the basolateral pdz protein scribble. Nature.

[B19] Bilder D, Li M, Perrimon N (2000). Cooperative regulation of cell polarity and growth by drosophila tumor suppressors. Science.

[B20] Dawid IB, Breen JJ, Toyama R (1998). Lim domains: Multiple roles as adapters and functional modifiers in protein interactions. Trends Genet.

[B21] Bach I (2000). The lim domain: Regulation by association. Mech Dev.

[B22] Yi J, Kloeker S, Jensen CC, Bockholt S, Honda H, Hirai H, Beckerle MC (2002). Members of the zyxin family of lim proteins interact with members of the p130cas family of signal transducers. J Biol Chem.

[B23] Nourry C, Grant SG, Borg JP (2003). Pdz domain proteins: Plug and play!. Sci STKE.

[B24] Cuppen E, Gerrits H, Pepers B, Wieringa B, Hendriks W (1998). Pdz motifs in ptp-bl and ril bind to internal protein segments in the lim domain protein ril. Mol Biol Cell.

[B25] Hung AY, Sheng M (2002). Pdz domains: Structural modules for protein complex assembly. J Biol Chem.

[B26] Songyang Z, Fanning AS, Fu C, Xu J, Marfatia SM, Chishti AH, Crompton A, Chan AC, Anderson JM, Cantley LC (1997). Recognition of unique carboxyl-terminal motifs by distinct pdz domains. Science.

[B27] Pistor S, Chakraborty T, Niebuhr K, Domann E, Wehland J (1994). The acta protein of listeria monocytogenes acts as a nucleator inducing reorganization of the actin cytoskeleton. Embo J.

[B28] Bubeck P, Pistor S, Wehland J, Jockusch BM (1997). Ligand recruitment by vinculin domains in transfected cells. J Cell Sci.

[B29] Borg JP, Marchetto S, Le Bivic A, Ollendorff V, Jaulin-Bastard F, Saito H, Fournier E, Adelaide J, Margolis B, Birnbaum D (2000). Erbin: A basolateral pdz protein that interacts with the mammalian erbb2/her2 receptor. Nat Cell Biol.

[B30] Apperson ML, Moon IS, Kennedy MB (1996). Characterization of densin-180, a new brain-specific synaptic protein of the o-sialoglycoprotein family. J Neurosci.

[B31] Saito H, Santoni MJ, Arsanto JP, Jaulin-Bastard F, Le Bivic A, Marchetto S, Audebert S, Isnardon D, Adelaide J, Birnbaum D, Borg JP (2001). Lano, a novel lap protein directly connected to maguk proteins in epithelial cells. J Biol Chem.

[B32] Nakagawa S, Huibregtse JM (2000). Human scribble (vartul) is targeted for ubiquitin-mediated degradation by the high-risk papillomavirus e6 proteins and the e6ap ubiquitin-protein ligase. Mol Cell Biol.

[B33] Legouis R, Gansmuller A, Sookhareea S, Bosher JM, Baillie DL, Labouesse M (2000). Let-413 is a basolateral protein required for the assembly of adherens junctions in caenorhabditis elegans. Nat Cell Biol.

[B34] Santoni MJ, Pontarotti P, Birnbaum D, Borg JP (2002). The lap family: A phylogenetic point of view. Trends Genet.

[B35] Albertson R, Doe CQ (2003). Dlg, scrib and lgl regulate neuroblast cell size and mitotic spindle asymmetry. Nat Cell Biol.

[B36] Brumby AM, Richardson HE (2003). Scribble mutants cooperate with oncogenic ras or notch to cause neoplastic overgrowth in drosophila. Embo J.

[B37] Pagliarini RA, Xu T (2003). A genetic screen in drosophila for metastatic behavior. Science.

[B38] Mathew D, Gramates LS, Packard M, Thomas U, Bilder D, Perrimon N, Gorczyca M, Budnik V (2002). Recruitment of scribble to the synaptic scaffolding complex requires guk-holder, a novel dlg binding protein. Curr Biol.

[B39] Roche JP, Packard MC, Moeckel-Cole S, Budnik V (2002). Regulation of synaptic plasticity and synaptic vesicle dynamics by the pdz protein scribble. J Neurosci.

[B40] Ganguly I, Mackay TF, Anholt RR (2003). Scribble is essential for olfactory behavior in drosophila melanogaster. Genetics.

[B41] Nguyen MM, Nguyen ML, Caruana G, Bernstein A, Lambert PF, Griep AE (2003). Requirement of pdz-containing proteins for cell cycle regulation and differentiation in the mouse lens epithelium. Mol Cell Biol.

[B42] Ali SH, DeCaprio JA (2001). Cellular transformation by sv40 large t antigen: Interaction with host proteins. Semin Cancer Biol.

[B43] Montcouquiol M, Rachel RA, Lanford PJ, Copeland NG, Jenkins NA, Kelley MW (2003). Identification of vangl2 and scrb1 as planar polarity genes in mammals. Nature.

[B44] Murdoch JN, Henderson DJ, Doudney K, Gaston-Massuet C, Phillips HM, Paternotte C, Arkell R, Stanier P, Copp AJ (2003). Disruption of scribble (scrb1) causes severe neural tube defects in the circletail mouse. Hum Mol Genet.

[B45] Legouis R, Jaulin-Bastard F, Schott S, Navarro C, Borg JP, Labouesse M (2003). Basolateral targeting by leucine-rich repeat domains in epithelial cells. EMBO Rep.

[B46] Audebert S, Navarro C, Nourry C, Chasserot-Golaz S, Lecine P, Bellaiche Y, Dupont JL, Premont RT, Sempere C, Strub JM, Van Dorsselaer A, Vitale N, Borg JP (2004). Mammalian scribble forms a tight complex with the betapix exchange factor. Curr Biol.

[B47] Hirota T, Morisaki T, Nishiyama Y, Marumoto T, Tada K, Hara T, Masuko N, Inagaki M, Hatakeyama K, Saya H (2000). Zyxin, a regulator of actin filament assembly, targets the mitotic apparatus by interacting with h-warts/lats1 tumor suppressor. J Cell Biol.

[B48] Wang Y, Dooher JE, Koedood Zhao M, Gilmore TD (1999). Characterization of mouse trip6: A putative intracellular signaling protein. Gene.

[B49] Schaefer BC (1995). Revolutions in rapid amplification of cdna ends: New strategies for polymerase chain reaction cloning of full-length cdna ends. Anal Biochem.

[B50] Verschueren K, Remacle JE, Collart C, Kraft H, Baker BS, Tylzanowski P, Nelles L, Wuytens G, Su MT, Bodmer R, Smith JC, Huylebroeck D (1999). Sip1, a novel zinc finger/homeodomain repressor, interacts with smad proteins and binds to 5'-cacct sequences in candidate target genes. J Biol Chem.

[B51] Gietz RD, Woods RA (2002). Transformation of yeast by lithium acetate/single-stranded carrier DNA/polyethylene glycol method. Methods Enzymol.

[B52] Sadowski I, Bell B, Broad P, Hollis M (1992). Gal4 fusion vectors for expression in yeast or mammalian cells. Gene.

[B53] Buchert M, Schneider S, Adams MT, Hefti HP, Moelling K, Hovens CM (1997). Useful vectors for the two-hybrid system in mammalian cells. Biotechniques.

